# Wolf-Hirschhorn Syndrome-Associated Genes Are Enriched in Motile Neural Crest Cells and Affect Craniofacial Development in *Xenopus laevis*

**DOI:** 10.3389/fphys.2019.00431

**Published:** 2019-04-12

**Authors:** Alexandra Mills, Elizabeth Bearce, Rachael Cella, Seung Woo Kim, Megan Selig, Sangmook Lee, Laura Anne Lowery

**Affiliations:** Biology Department, Boston College, Chestnut Hill, MA, United States

**Keywords:** craniofacial development, developmental disorders, Wolf-Hirschhorn Syndrome, WHSC1, WHSC2, LETM1, TACC3, neural crest

## Abstract

Wolf-Hirschhorn Syndrome (WHS) is a human developmental disorder arising from a hemizygous perturbation, typically a microdeletion, on the short arm of chromosome four. In addition to pronounced intellectual disability, seizures, and delayed growth, WHS presents with a characteristic facial dysmorphism and varying prevalence of microcephaly, micrognathia, cartilage malformation in the ear and nose, and facial asymmetries. These affected craniofacial tissues all derive from a shared embryonic precursor, the cranial neural crest (CNC), inviting the hypothesis that one or more WHS-affected genes may be critical regulators of neural crest development or migration. To explore this, we characterized expression of multiple genes within or immediately proximal to defined WHS critical regions, across the span of craniofacial development in the vertebrate model system *Xenopus laevis*. This subset of genes, *whsc1*, *whsc2*, *letm1*, and *tacc3*, are diverse in their currently-elucidated cellular functions; yet we find that their expression demonstrates shared tissue-specific enrichment within the anterior neural tube, migratory neural crest, and later craniofacial structures. We examine the ramifications of this by characterizing craniofacial development and neural crest migration following individual gene depletion. We observe that several WHS-associated genes significantly impact facial patterning, cartilage formation, neural crest motility *in vivo* and *in vitro*, and can separately contribute to forebrain scaling. Thus, we have determined that numerous genes within and surrounding the defined WHS critical regions potently impact craniofacial patterning, suggesting their role in WHS presentation may stem from essential functions during neural crest-derived tissue formation.

## Introduction

Wolf-Hirschhorn Syndrome (WHS) is a developmental disorder characterized by intellectual disability, delayed pre- and post-natal growth, heart and skeletal defects, and seizures (Hirschhorn et al., 1965; Wolf et al., 1965; Zollino et al., 2000; Battaglia et al., 2015). A common clinical marker of WHS is the “Greek Warrior Helmet" appearance; a facial pattern with a characteristic wide and flattened nasal bridge, a high forehead, prominent eyebrow arches and pronounced brow bones, hypertelorism (widely spaced eyes), a short philtrum (space between nose and lip), and micrognathia (undersized jaw). The majority of children with the disorder are microcephalic, and have abnormally positioned ears with underdeveloped cartilage. Comorbid midline deficits can occur, including cleft palate and facial asymmetries (Battaglia et al., 2015).

Craniofacial malformations are one of the most prevalent forms of congenital defects (Gorlin et al., 2001; Trainor, 2010), and can significantly complicate palliative care and quality of life (Merrow, 2016). Given the commanding role of cranial neural crest (CNC) cells in virtually all facets of craniofacial patterning, craniofacial abnormalities are typically attributable to aberrant CNC development (Walker and Trainor, 2006; Trainor, 2010). A striking commonality in the tissues that are impacted by WHS is that a significant number derive from the CNC. Despite this, little is known about how the vast diversity of genetic disruptions that underlie WHS pathology can contribute to craniofacial malformation, and no study has sought to characterize impacts of these genotypes explicitly on CNC behavior.

**FIGURE 1:**
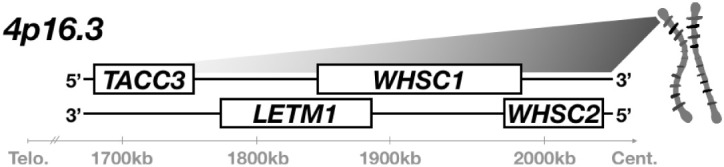
WHS is typically caused by heterozygous microdeletion of numerous genes within 4p16.3. A segment of this region is illustrated here. A microdeletion that spans at least *WHSC1, WHSC2*, and *LETM1* is currently assumed to be necessary for full WHS diagnostic presentation; children affected by the disorder often possess larger deletions that extend further telomeric and impact additional genes, such as *TACC3*.

WHS is typically caused by small, heterozygous deletions on the short-arm of chromosome 4 (4p16.3) (Figure 1), which can vary widely in position and length. Initially, deletion of a very small critical region, only partial segments of two genes, was thought to be sufficient for full syndromic presentation (Gandelman et al., 1992; Wright et al., 1997; Stec et al., 1998; Bergemann et al., 2005). These first putative associated genes were appropriately denoted as Wolf-Hirschhorn Syndrome Candidates 1 and 2 (*WHSC1, WHSC2*) (Wright et al., 1997; Stec et al., 1998; Rauch et al., 2001; Simon and Bergemann, 2008; Nimura et al., 2009; Ahmed et al., 2015). However, children with WHS largely demonstrate 4p disruptions that impact not only this intergenic region between *WHSC1* and *WHSC2*, but instead affect multiple genes both telomeric and centromeric from this locus (Zollino et al., 2003). Focus was drawn to these broader impacted regions when cases were identified that neglected this first critical region entirely but still showed either full or partial WHS presentation, prompting the expansion of the originally defined critical region to include a more telomeric segment of *WHSC1*, and a new candidate, LETM1 (Zollino et al., 2000; South et al., 2008). These discrepancies are increasingly rectified by mounting evidence that true cases of the syndrome are multigenic (Zollino et al., 2008; Andersen et al., 2014; Battaglia et al., 2015); the disorder can arise from numerous and varied microdeletions, but no singular gene depletion appears sufficient to drive its full presentation.

This multigenic nature gains new layers of complexity in the context of the functional diversity of WHS’s affected genes. The first described gene in WHS, *WHSC1*, encodes for a global histone methyltransferase (Nimura et al., 2009). *WHSC2* encodes the protein Negative Elongation Factor A (NELF-A), which has multiple DNA binding motifs, and has been shown to interact with pre-mRNAs and to inhibit RNA polymerase II activity (Mariotti et al., 2000; Kerzendorfer et al., 2012; Battaglia et al., 2015). Leucine zipper and EF-hand containing transmembrane protein (LETM1*)* plays a role as a mitochondrial ion transporter (Schlickum et al., 2004). Transforming acidic coiled-coil protein 3 (TACC3) is a cytoskeletal regulator which facilitates microtubule growth in multiple embryonic cells (Nwagbara et al., 2014), in addition to critical functions in microtubule elongation at the mitotic spindle (Gergely et al., 2000; Peset and Vernos, 2008; Cheeseman et al., 2011; Ha et al., 2013a,b). Few concrete links can be made between these gene products to speculate how or why their collective depletion is required to produce full WHS pathology.

Our emerging understanding of WHS as a multigenic developmental disorder necessitates its study as such – with a renewed focus on how the depletion of these genes combinatorially contribute to a collaborative phenotype. However, a central problem arises that entirely precludes this effort: we largely lack a fundamental understanding of how singular WHS-affected genes function in basic developmental processes. Furthermore, animal models of WHS-associated gene depletion have occurred across numerous species and strains, with no unifying model to offer a comparative platform. Given the disorder’s consistent and extensive craniofacial malformations, it seems especially prudent to establish whether these genes serve critical functions explicitly during processes governing craniofacial morphogenesis.

To better understand the role of these four genes in WHS pathogenesis, we examined the contributions of *whsc1*, *whsc2*, *letm1*, and *tacc3* to early craniofacial patterning in *Xenopus laevis*. We first examined expression profiles of these transcripts across early embryonic development, and notably, observed enrichment of all four transcripts in motile CNCs of the pharyngeal arches, which invites the hypothesis that they may impact neural crest development and migration. Knockdown (KD) strategies were then utilized to examine WHS-associated gene contributions to facial morphogenesis and cartilage development. We find that all KDs could variably affect facial morphology. Perhaps most notably, Whsc1 depletion increased facial width along the axis of the tragion (across the eyes or temples), recapitulating one feature of WHS craniofacial malformation. We performed both *in vivo* and *in vitro* CNC migration assays that illustrate that Whsc1 and Tacc3 can directly affect pharyngeal arch morphology and CNC motility rates. Separately, as most of the examined transcripts also demonstrated enrichment in the anterior neural tube, we examined their impacts on embryonic forebrain scaling. We found that depletion of three of the four genes could additionally impact forebrain size. Together, our results support a hypothesis that WHS produces consistent craniofacial phenotypes (despite a vast diversity in genetic perturbations), in part due to numerous genes within the affected 4p locus performing critical and potentially combinatorial roles in neural crest migration, craniofacial patterning, cartilaginous tissue formation, and brain development. Furthermore, this work is the first to perform depletion of multiple WHS-affected genes on a shared, directly-comparable background, laying an essential foundation for future efforts to model, integrate, or predict interactions of diverse genetic disruptions within the context of a multigenic syndrome.

## Results

### Numerous WHS-Affected Genes Demonstrate Enriched Expression in the Pharyngeal Arches, Early Nervous System, and Embryonic Craniofacial Structures

Pronounced and characteristic craniofacial dysmorphism is one of the most recognizable features of WHS-affiliated 4p16.3 microdeletions. Children with the disorder demonstrate a low-profile nasal bridge and prevalent lower forehead, with wide-set eyes and a short philtrum (together commonly referred to as the Greek Warrior’s Helmet presentation). Microcephaly and micrognathia are present with varying severity, and comorbidities commonly include facial asymmetries and cleft palate (Paradowska-Stolarz, 2014). Given the commanding role of CNC cell proliferation, migration, and differentiation in properly coordinated facial patterning of nearly all of these affected tissues, we hypothesized that certain WHS-affected genes could play critical roles in neural crest maintenance, motility, or specification, and that their depletion would thus disproportionately impact tissues derived from the neural crest.

**FIGURE 2:**
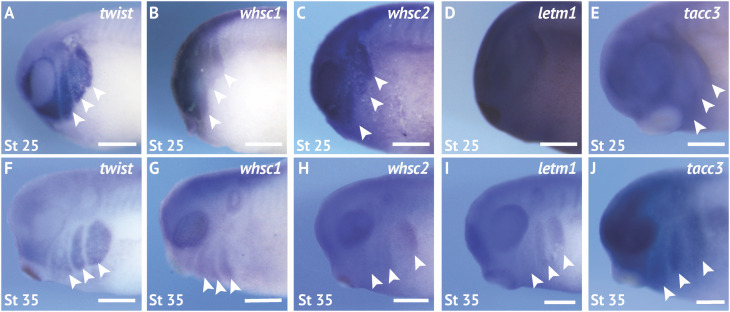
WHS related genes are expressed in the migrating neural crest cells during embryonic development. **(A,F)** Lateral views of whole mount *in situ* hybridizations for *twist*, a CNC-enriched transcription factor. Arrows indicate the pharyngeal arches (PA). **(B–E,G–J)** *In situ* hybridizations for *whsc1*, *whsc2*, *letm1*, and *tacc3* demonstrate enrichment in CNCs that occupy the PAs (*n* = 20 per probe, per timepoint). Scalebar is 250 μm.

We first performed coordinated examinations of spatiotemporal expression of commonly affected genes in the 4p16.3 locus across craniofacial development. To this end, we performed *in situ* hybridization with DIG-labeled antisense RNA probes against four genes within and proximal to the last defined WHS critical region (*whsc1*, *whsc2*, *letm1*, and *tacc3*) (Figure 1 and Supplementary Figure S1; for *in situ* hybrization controls against mRNA sense strands, see Supplementary Figure S1Y). During early craniofacial morphogenesis at stage 25, we note enriched expression of *whsc1*, *whsc2*, and *tacc3* in the migratory CNCs that populate the pharyngeal arches (Figure 2B,C,E). Their enrichment closely resembles the expression pattern of the CNC-enriched transcription factor *twist* (Figure 2A,F). Comparatively, *letm1* (Figure 2D) demonstrates ubiquitous expression. Interestingly, with the exception of *tacc3*, these transcripts are not significantly enriched in specified, premigratory neural crest (st. 16) (Supplementary Figure S1). By stage 35, all four transcripts are enriched in pharyngeal arches (Figure 2G–J); *letm1* expression appears to reduce in neighboring tissues, while remaining selectively enriched in CNCs during later stages of migration within the pharyngeal arches (Figure 2I). There is also significant transcription of all four genes within the anterior neural tube. Later in tailbud stages, some transcripts maintain enriched expression in the forebrain, most notably *whsc2*; meanwhile, *whsc1*, *whsc2*, and *letm1* are enriched in tissues within the head and face (Supplementary Figures S1E,F,K,L,Q,R,W,X). Additionally, *whsc1*, *letm1*, and *tacc3* expression show potential overlap with cardiac tissue (Supplementary Figures S1E,Q,W).

### WHS-Affected Genes Are Critical for Normal Craniofacial Morphology

**FIGURE 3:**
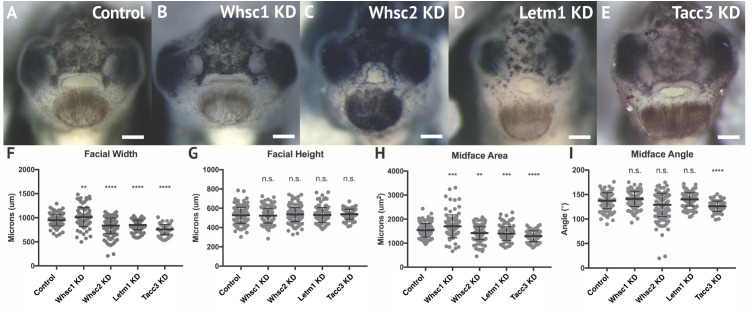
WHS related gene depletion affects craniofacial morphology. **(A–E)** Frontal views of 3dpf embryos (st. 40) following WHS gene single KD. **(F–I)** Measurements for facial width, height, midface area, and midface angle. A significant 6.54% increase in facial width and 11.43% increase in midface area were observed for Whsc1 KD. Whsc2 KD caused a 12.01% reduction in facial width and a 6.79% reduction in midface area. Letm1 KD caused a 10.33% decrease in facial width and a 8.49% decrease in midface area. Tacc3 KD caused a 21.27% decrease in facial width and a 16.33% decrease in midface area, and an 8.27% decrease in midface angle. Significance determined using a student’s unpaired *t*-test. (Embryos quantified: Control = 137, Whsc1 KD = 100, Whsc2 KD = 185, Letm1 KD = 115, Tacc3 KD = 79.) ^∗∗∗∗^*P* < 0.0001, ^∗∗∗^*P* < 0.001, ^∗∗^*P* < 0.01, n.s., not significant. Scalebar = 250 μm.

Given that all four genes showed enrichment in migratory neural crest by stage 35, and most demonstrated enduring transcription in later craniofacial tissues, we hypothesized that their protein products may function in craniofacial morphogenesis. To this end, we performed partial genetic depletions of WHS-associated genes in *X. laevis* embryos (Supplementary Figure S2), followed by morphometric analyses of craniofacial landmarks between WHS-associated gene KD and control conditions from the same clutch (Figure 3). Measurements to quantify facial width, height, midface area, and midface angle were performed as previously described (Kennedy and Dickinson, 2014) at stage 40 (Supplementary Figure S3).

Individual depletion of the examined WHS-affected genes demonstrated pronounced impacts on facial patterning (Figure 3A–E). Whsc1 depletion significantly increased facial width (Figure 3F), and this increase accompanied a significant increase in facial area (Figure 3H). Whsc2, Letm1, and Tacc3 depletion conversely narrowed facial width at this axis (Figure 3F), and additionally decreased facial area (Figure 3H). None of these changes were proportional to facial height, which was unaffected by gene depletion. In nearly all cases, the distribution of facial features was normal. Only Tacc3 depletion modestly affected the mid-face angle, a parameter describing the relationship between the eyes and mouth (Figure 3I). Importantly, all facial phenotypes could be rescued by co-injection with full-length mRNA transcripts of their targets (Supplementary Figure S4), indicating that phenotypes were specific to WHS-associated gene depletion. Taken together, these results are consistent with a possibility that Whsc1 depletion may be sufficient to drive frontonasal dysmorphism, while Whsc2, Letm1, and Tacc*3* depletions may contribute to complex or epistatic interactions, or mediate additional characteristic facial features of the disorder.

### WHS-Affected Genes Maintain Craniofacial Cartilage Size and Scaling

**FIGURE 4:**
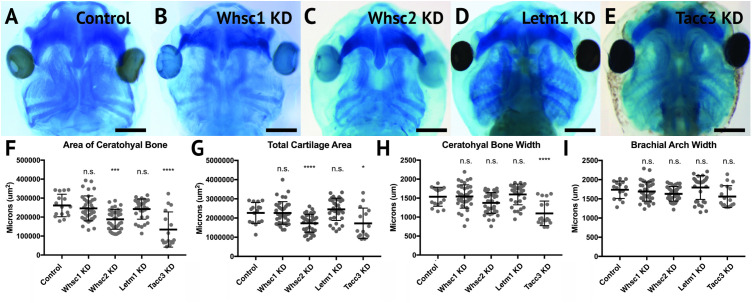
Knockdown of Whsc2 and Tacc3 impact cartilage morphology. **(A–E)** Ventral view of 6dpf embryos following single WHS-assoc. gene KD, stained with Alcian Blue to label cartilage elements. **(F–I)** Measurements of the average area and width of the ceratohyal cartilage, total cartilage area, and width of the brachial arches. Neither Whsc1 nor Letm1 KD caused a significant change in any measured parameter. Whsc2 KD caused a 27.94% decrease in average area of the ceratohyal cartilage, and a 23.87% decrease in area of all craniofacial cartilage. Tacc3 KD caused a 48.5% decrease in the average area of the ceratohyal cartilage, a 24.03% decrease in total cartilage area, and a 28.58% decrease in ceratohyal cartilage width. Significance was determined using a student’s unpaired *t*-test. (Embryos quantified: Control = 17, Whsc1 KD = 41, Whsc2 KD = 39, Letm1 KD = 34, Tacc3 KD = 11.) ^∗∗∗∗^P < 0.0001, ^∗∗∗^*P* < 0.001, ^∗^*P* < 0.05, n.s., not significant. Scalebar is 250μm.

A majority of WHS cases demonstrate defects in cartilage and skeletal formation. Notable examples include underdeveloped ears with reduced or missing cartilage, micrognathia, tooth malformation, short stature, and delayed growth of the head and body (Zollino et al., 2008; Battaglia et al., 2015), as well as jaw and throat malformations that significantly impair speech, feeding, and swallowing (Battaglia et al., 2015). The etiology of these co-morbidities is virtually unknown. As craniofacial cartilage and bone are largely derived from the CNC (Trainor and Andrews, 2013), we hypothesized that one or more of these genes may play a critical role in craniofacial cartilage formation. To test this, we performed depletion of Whsc1, Whsc2, Letm1, and Tacc3 as described above, in order to survey their impact on scaling and morphology of craniofacial cartilage in *X. laevis* larvae (Figure 4A–I).

Depletion of either Whsc2 or Tacc3 was sufficient to reduce the combined area of the ceratohyal and branchial arch cartilages (CH and BR, respectively, Figure 4A), in 6 days (stage 47) embryos (Figure 4I). These effects were also explicitly shown in the ceratohyal area alone (Figure 4F). Ceratohyal cartilage width was also reduced upon Tacc3 depletion (Figure 4G). Somewhat surprisingly, given the impact of Whsc1 depletion on facial width, its depletion did not increase ceratohyal width or area. Similarly, Letm1 depletion did not reduce cartilage area, despite reduction in overall facial width. These results indicate that *whsc2* and *tacc3*, genes both within and immediately proximal to the critically-affected locus of WHS, are critical for early cartilaginous tissue formation, illustrating a potential avenue through which larger human 4p deletions may exacerbate phenotypic severity. Importantly, these effects are demonstrable at 6d post-fertilization, suggesting that early partial depletion of these transcripts produces lingering impacts on craniofacial patterning (first measured at 3 days post-fertilization, Figure 3) that are not ameliorated later in development. We hypothesized that these persistent patterning defects following early depletion of WHS-associated genes may then arise indirectly, from impacts on their embryonic progenitors.

### *Whsc1* and *tacc3* Are Critical for Normal Pharyngeal Arch Morphology and Cranial Neural Crest Cell Motility

Given the enrichment of WHS-affected gene transcripts in CNCs during stages that correspond with their migration (st. 25–35), we hypothesized that their depletion may directly compromise CNC motility. To examine this, we used single-hemisphere injection strategies to generate left-right chimeric embryos (for work flow, see Supplementary Figure S5), and internally compared patterns of *twist* expression to track the progress of migrating CNC along control or depleted sides.

**FIGURE 5:**
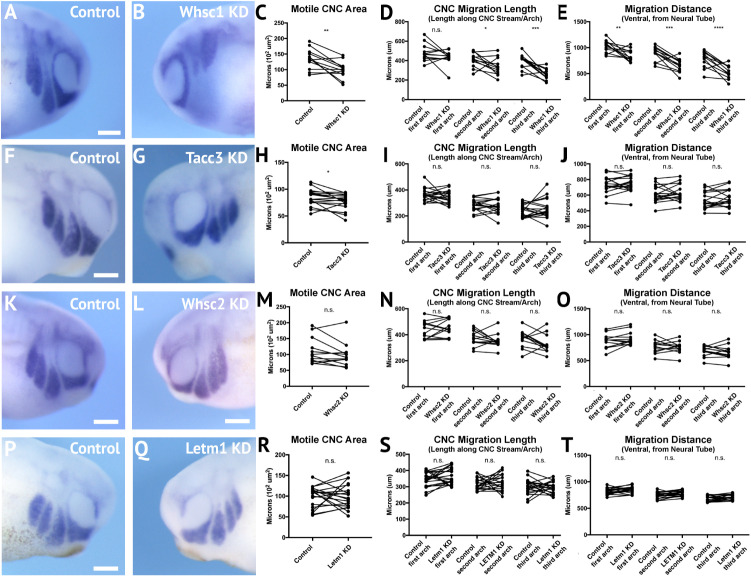
Knockdown of Whsc1 and Tacc3 decrease CNC migration *in vivo*. **(A,B,F,G,K,L,P,Q)** Anterior lateral views of tailbud stage embryos (depicted at st. 27), following whole mount *in situ* hybridization against *twist*. Each column of panels **(A,B,F,G,K,L,Q)** are lateral views of two sides of the same embryo. **(C–E,H–J,M–O,R–T)** Measurements were taken for the total area of the three PA (Arch 1-3 extend anterior to posterior), the length of each individual arch, and the migration distance, as measured from the dorsal most tip of each arch to the neural tube. Embryos were stained and quantified at stages 25–30. **(K–T)** Letm1 or Whsc2 KD did not significantly affect any of the measured parameters. **(F–J)** Tacc3 KD expression caused an 8.33% decrease in the total PA area, but did not affect length or arch migration. **(A–E)** Whsc1 KD caused a 23.57% decrease in PA area. Additionally, the length of the second and third pharyngeal arches decreased by 14.72 and 31.70%, respectively. The migration distance of the first, second and third pharyngeal arches decreased by 15.75, 24.04, and 29.29%, respectively. Significance determined using a student’s paired *t*-test. (Embryos quantified: Whsc1 KD = 13, Tacc3 KD = 18, Whsc2 KD = 12, Letm1 KD = 19.) ^∗∗∗∗^*P* < 0.0001, ^∗∗∗^*P* < 0.001, ^∗^*P* < 0.05, n.s., not significant. Scalebar is 250 μm.

Following single-sided Whsc1, Whsc2, Letm1, or Tacc3 depletion, embryos were staged to 25–30, fixed, and *in situ* hybridization was performed against *twist*. Measurements of length and area of *twist* expression were taken, to quantify CNC migration away from the anterior neural tube, and these were compared to their internal, contralateral controls. Whsc1 and Tacc3 depletion reduced total area of CNC streams (Figure 5C,H). Further, when Whsc1 levels were reduced, the CNC streams were shorter in length (Figure 5D), and their ventral migration distance was reduced compared to paired controls (Figure 5E). Whsc2 and Letm1 reduction, in contrast, did not result in any significant changes to CNC migration. This suggests a role specifically for *whsc1* and *tacc3* in maintaining normal CNC motility into the PAs.

**FIGURE 6:**
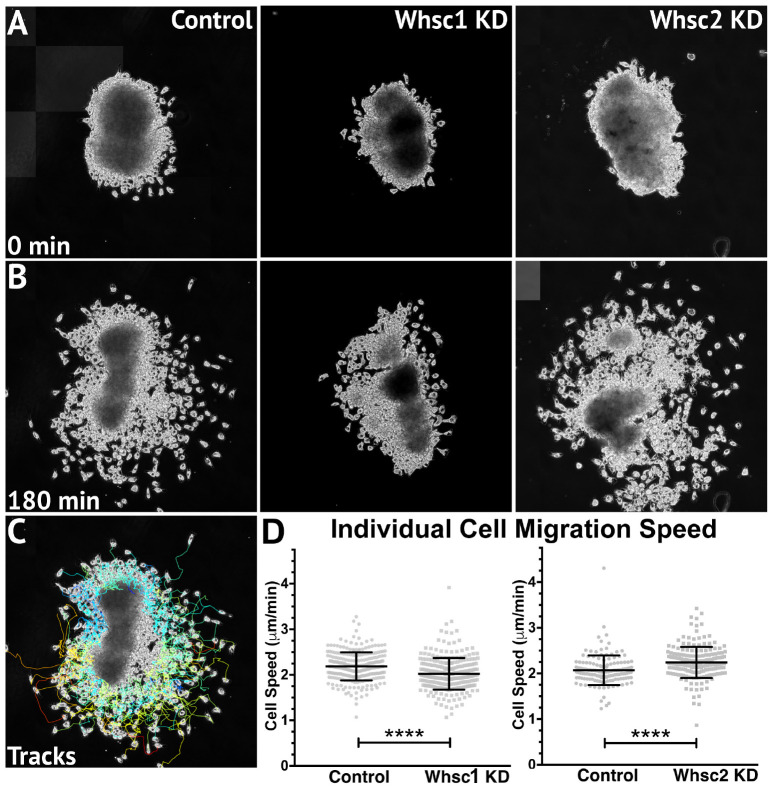
Whsc1 manipulation alters CNC migration speeds *in vitro*. Dissected CNC explants from control, Whsc1 KD, or Whsc2 KD embryos were plated on fibronectin-coated coverslips, allowed to adhere and begin migration, and imaged for 3 h using 20× phase microscopy. **(A)** Representative explants at initial timepoint (0 min). **(B)** Explants after 3 h migration time. **(C)** Representative tracks generated by FiJi Trackmate plug-in. **(D)** Mean track speeds of Whsc1 or Whsc2 KD explants compared to their controls. (Explants quantified: 3–4 explants from control and KD embryos were plated for each experiment, explants with neural or epithelial contaminant were excluded from analysis. Three separate experiments were performed for each depletion. Whsc1 controls: 272 cells, 9 explants. Whsc1 KD: 282 cells, 9 explants. Whsc2 controls: 151 cells, 12 explants. Whsc2 KD: 195 cells, 8 explants.) ^∗∗∗∗^*P* < 0.0001, n.s., not significant. Scalebar is 250μm.

Smaller areas of *twist* expression, as shown with either Whsc1 or Tacc3 depletion, could result from reduced migration rates, as cells accumulate into denser, slower packs; but it is also possible that CNCs may occupy smaller regions if a genetic perturbation affects their proliferation rates (resulting in fewer cells overall). To determine whether Whsc1 depletion could specifically impact neural crest migration speed, *in vitro* migration assays were performed as described previously (Alfandari et al., 2001; Milet and Monsoro-Burq, 2014). Whole embryos were injected with either control or Whsc1 KD strategies, and their CNCs were dissected prior to delamination from the neural tube (st. 17). These tissue explants were cultured on fibronectin-coated coverslips, and trajectories of individual cells that escaped the explant were mapped using automated particle tracking (Schindelin et al., 2012; Tinevez et al., 2017). Whsc1 depletion resulted in slower individual cell speeds compared to controls (Figure 6B–D and Supplementary Video S1). Tacc3 KD was also sufficient to reduce individual CNC speeds (not shown). We then compared these results to those obtained following Whsc2 depletion. As Whsc2 KD was not sufficient to alter CNC streaming areas *in vivo* (Figure 5K–O), we hypothesized that cell motility speed *in vitro* would not be affected by this depletion. Instead, Whsc2 KD resulted in a significant increase in speed of CNCs migrating in culture (Figure 6D). As CNC migration is heavily restricted *in vivo* due to repellent and non-permissive substrate boundaries within the Pas (Szabó et al., 2016), in addition to the coordinated relationships between CNC and placodal cell migration (Theveneau et al., 2013), it is not surprising that a moderate increase in individual cell motility speeds *in vitro* may not correspond to a notable increases in CNC streaming within the PAs. In contrast, a deficit in individual cell migration rate, as shown with Whsc1 and Tacc3 depletion, may lack compensatory strategies and more directly delay CNC streaming. Thus, we show that Whsc1 depletion alters CNC infiltration into the PAs, and that this effect could be directly driven by a reduction in individual CNC migration rates.

### WHS-Related Genes Impact Forebrain Morphology

In addition to craniofacial dysmorphism, children with 4p16.3 microdeletions demonstrate mild to profound intellectual disability, with a large majority displaying significant psychomotor and language delays that entirely preclude effective communication (Zollino et al., 2008; Battaglia et al., 2015; Bernardini et al., 2018). Larger microdeletions have generally been correlated to more severe intellectual disability and microcephaly, implying that numerous WHS-affected genes may function combinatorially or synergistically to facilitate central nervous system development and cognitive function (Zollino et al., 2008). Alternatively, this may suggest that genes that are further telomeric within the affected loci could be more impactful contributors to cognitive deficits.

We have largely focused our current efforts to examine the developmental contributions of WHS-affected genes to neural crest migration and craniofacial development, and development of the central nervous system should largely be considered to function distinctly and be examined in future works. However, given the significant craniofacial malformations demonstrated with WHS-associated gene depletion, and the intimate ties between central nervous system and craniofacial development (Demyer et al., 1964; Aoto and Trainor, 2015), we also performed initial characterization of how these WHS-affected genes may singularly contribute to one aspect of neurodevelopment; embryonic forebrain scaling.

**FIGURE 7:**
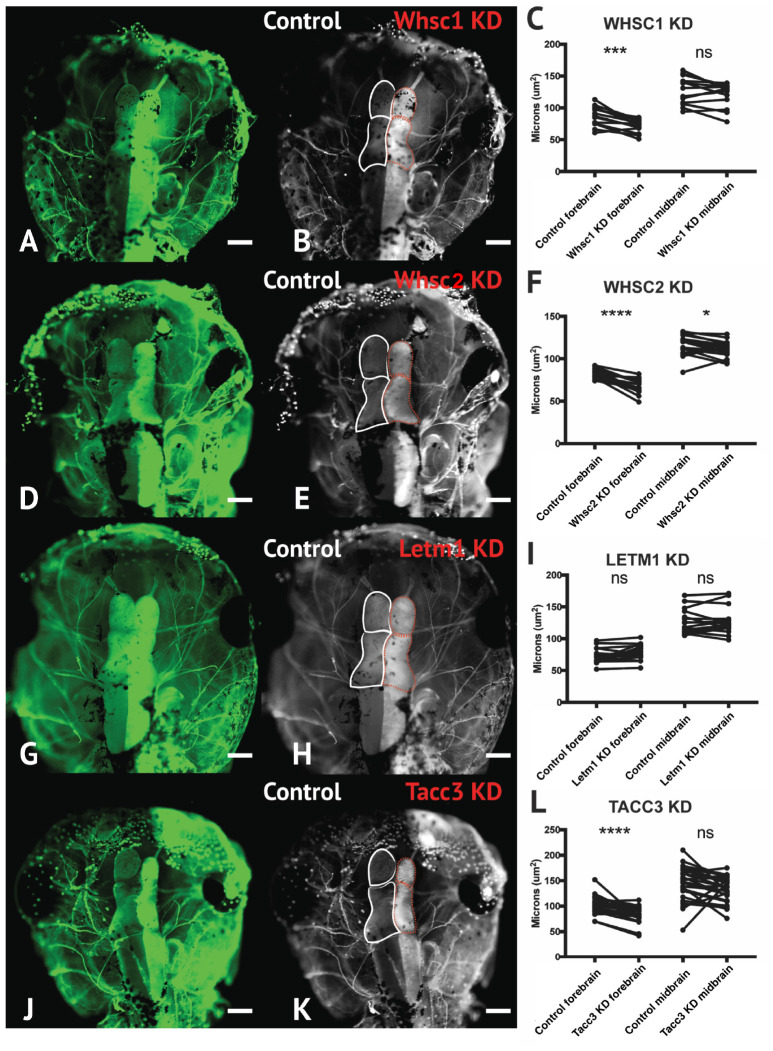
Whsc1, whsc2, and tacc3 facilitate normal forebrain development. **(A,B,D,E,G,H,J,K)** Dorsal view of *X. laevis* half-embryo gene depletions (6 days post-fertilization), following alpha-tubulin immunolabeling to highlight nervous system. **(B,E,H,K)** Dorsal view of embryos with superimposed outlines of forebrain and midbrain structures. Internal control is on left (white), depleted side is on right (dashed red). (Alpha-tubulin staining is bilateral; exogenous eGFP on KD side persisted in embryos shown, causing a unilaterally enriched green signal.) **(C,F,I,L)** Area of forebrain and midbrain. Whsc1 KD reduced forebrain area by 17.65%. Whsc2 KD reduced forebrain area by 17.33% and midbrain area by 4.14%. Letm1 KD caused no significant change in brain size. Tacc3 KD caused a 16.05% decrease in forebrain area. Significance determined using a student’s paired *t*-test. (Embryos quantified: Whsc1 KD = 14, Whsc2 KD = 18, Letm1 KD = 12, Tacc3 KD = 26.) ^∗∗∗∗^*P* < 0.0001, ^∗∗∗^*P* < 0.001, ^∗^*P* < 0.05, n.s., not significant. Scalebar is 250 μm.

To address the impact of Whsc1, Whsc2, Letm1, and Tacc3 on forebrain size, we performed half-embryo depletions as above, and examined the outcomes on embryonic brain size. Embryos were injected with single-hemisphere depletion strategies at the 2-cell stage, and then allowed to mature to 6 days (st. 47) prior to fixation. Immunolabeling for alpha-tubulin was carried out to highlight neuronal morphology (Figure 7; for experimental workflow, see Supplementary Figure S5), and brain areas were compared using paired *t*-tests between KD and control hemispheres. Forebrain size was significantly reduced with Whsc1, Whsc2, or Tacc3 KD (Figure 7B,C,E,F,K,L). Control injections did not affect brain size, relative to internal non-injected controls (Supplementary Figure S5). Whsc2 depletion caused an additional decrease to midbrain area (Figure 7F). Letm1 depletion did not impact forebrain sizing (Figure 7G–I), however, *LETM1* deletion is suspected to be the major contributor to seizure development in children with the disorder (Jiang et al., 2009; Andersen et al., 2014). This only highlights the importance of future characterizations of the cell biological functions of WHS-impacted genes, as it could be suggested that LETM1 depletion may instead disrupt normal neuronal excitation, connectivity, or survival (Huang et al., 2017). These initial investigations suggest that *whsc1*, *whsc2*, and *tacc3* facilitate normal forebrain development, and perhaps that their depletion is relevant to WHS-associated microcephaly.

## Discussion

**FIGURE 8:**
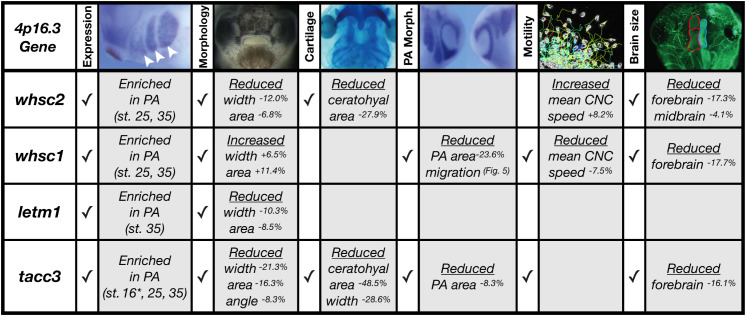
Partial depletion of WHS-affected genes demonstrates numerous impacts on craniofacial development and neural crest migration. Tissues are denoted as affected (checked box) if phenotypes were significantly different from control (*p* ≤ 0.05); see individual figures for data distribution and statistics. (Abbreviations: PA, Pharyngeal Arch) ^∗^Denotes pre-migratory CNC (st. 16).

We have shown that four genes frequently affected in WHS, a human genetic disorder stemming from a heterozygous microdeletion on the short arm of chromosome four, can contribute to normal craniofacial morphogenesis in *Xenopus laevis* (Figure 8). We also provide evidence that neural crest migration deficits may significantly contribute to the signature craniofacial dysmorphism of WHS. Specifically, we demonstrate, for the first time, that WHS-associated genes are enriched in motile neural crest and contribute to normal craniofacial patterning and cartilage formation (*whsc1*, *whsc2*, *letm1*, and *tacc3*). Two of these genes directly impact individual CNC cell migration (*whsc1* and *tacc3*), revealing new basic roles for these genes in embryonic development.

It is increasingly appreciated that full WHS presentation is multigenic (Zollino et al., 2008); case studies of children with singular gene depletions even in critical regions have historically demonstrated milder syndromic presentations that lack the full range of expected symptoms (intellectual disability, craniofacial abnormalities, seizures, and heart, skeletal, and urogenital defects) (Rauch et al., 2001). While we have narrowed our examinations to focus on how WHS-affected genes contribute to facial patterning, our findings align well with the idea that WHS presentation is a cumulative product of the impacted locus. While Whsc1 and Tacc3 depletions impacted all or nearly all examined aspects of craniofacial development at these stages, Whsc1 KD did not produce significant cartilage malformations in isolation, and Tacc3 KD narrowed and condensed facial features in a way that appears less analogous to the human “Greek Warrior Helmet” phenotypic presentation.

Of important note, then, only Whsc1 depletion appeared to cause hypertelorism, or facial widening at the level of the eyes and nasal bridge (Figure 3, 8). As the eyes correspond to the peripheral extrema of the tadpole face, this contributed to a wider face along the axis of the tragion, which may correlate with 3D morphological mapping data that demonstrates an overall facial widening in children with WHS along the same axis (Hammond et al., 2012). It is interesting to predict that normal WHSC1 levels could facilitate normal neural crest migration, and in a separate role more explicit to this tissue region, also limit inappropriate proliferation and expansion. In potential support of this, one of WHSC1’s more established roles is that of an H3K36 methyltransferase, an epigenetic regulator that has been billed as oncogenic, given high levels of dysregulation in some cancer tissues (Huang et al., 2013; Kuo et al., 2013), and its potential to orchestrate transcriptional programs that drive unchecked proliferation (Park et al., 2018). Other studies report its function to be that of a tumor suppressor, given its high mutation rate in lymphomas (Beà et al., 2013; Zhang et al., 2014); additionally, *whsc1* knockout or depletion in zebrafish demonstrated enlarged hearts, brains, and predisposition to swim bladder tumors (Yamada-Okabe et al., 2010; Yu et al., 2017), suggesting unchecked expansion of developmental progenitors. As this duality likely partially reflects differential regulation of WHSC1 behavior during development and in the context of oncogenesis, an explicit examination of how WHSC1 functions to regulate tissue expansion and development in the extreme anterior domain may be warranted (Jacox et al., 2016). Additionally, given that the other three WHS-affected genes instead narrowed facial width and area, this invites further investigation into how these depletions function combinatorially to generate the full signature of WHS craniofacial dysmorphism.

Within that effort, however, it is worthwhile to note that WHS-associated gene depletion in *X. laevis* almost certainly diverges from perfect recapitulation of WHS pathology. *Xenopus* has proven to be an invaluable model for the study of human craniofacial development and disorders (Kennedy and Dickinson, 2014; Tabler et al., 2014; Devotta et al., 2016; Dickinson, 2016; Liu, 2016; Deniz et al., 2017; Dubey and Saint-Jeannet, 2017; Griffin et al., 2018), given the highly conserved developmental pathways that drive neural crest migration, differentiation, and craniofacial morphogenesis between systems. Nonetheless, there are gross morphological differences that prevent some direct correlations. It is noteworthy that the CNC that give rise to the ceratohyal cartilage in *Xenopus* will later give rise to far anterior portions of the face, and combine with contributions from the Meckel’s cartilage to form some regions of the jaw (Gross and Hanken, 2008; Kerney et al., 2012), but equivalent human craniofacial structures undergo distinct development (Frisdal and Trainor, 2014). Loosely, the ceratohyal cartilage in *X. laevis* is formed from CNC of the second PA (Gross and Hanken, 2008; Dubey and Saint-Jeannet, 2017); which in human development will give rise to tissues of the hyoid (Frisdal and Trainor, 2014). Morphological impacts resulting from aberrant development of these tissues, as was shown with either Tacc3 or Whsc2 depletion (Figure 4), may then have more direct correlates to human WHS pathology in the context of aberrant pharyngeal development, rather than explicitly in jaw formation or WHS-associated micrognathia.

Our work has demonstrated consistent enrichment of WHS-associated genes in CNCs, and their necessity for normal formation of their derivatives, however, this largely neglects why any of these transcripts may be exceptionally critical in these tissues. This question must be left to some speculation; the precise cell biological roles of all WHS-affected genes warrant much more comprehensive study in the context of embryonic development and cell motility. We have previously summarized some of the known roles of these genes and how they may influence CNC development (Rutherford and Lowery, 2016), but a brief summary incorporating recent work is outlined here.

*WHSC2* encodes the gene product NELFA, which functions within the NELF complex to decelerate or pause RNA polymerase II activity (Luo et al., 2013). This pausing mechanism is thought to function as a means of synchronizing rapid or constitutive expression of specific transcripts (Gilchrist et al., 2010; Adelman and Lis, 2012; Pan et al., 2014). NELF complex components are required during early embryogenesis (Amleh et al., 2009), but their relevance in craniofacial morphogenesis and neural crest migration is entirely unknown. Recent work suggests the NELF complex facilitates cancer cell proliferation and motility, downstream of its regulation of cell-cycle control transcripts (El Zeneini et al., 2017). Given that motility and proliferation inherently compete for cytoskeletal machinery (Matus et al., 2015), the CNC’s somewhat unique need to undergo both rapid expansion and directed motility (Monsoro-Burq, 2015) within the same developmental stages may benefit from these additional levels of coordination, but this remains entirely speculative.

LETM1 localizes to the inner mitochondrial membrane (Schlickum et al., 2004), where it acts as a Ca2(+/H(+ anti-porter to regulate Ca2(+ signaling and homeostasis (Jiang et al., 2013)[72], which can directly affect activity of mitochondrial metabolic enzymes. LETM1 was shown to actively regulate pyruvate dehydrogenase activity, tying its roles directly to glucose oxidation (Durigon et al., 2018)[73]. Its ubiquitous enrichment across early development (Fig.Figure 2D), and enduring expression within motile CNC (Fig.Figure 2I) might suggest distinct and spatiotemporal metabolic needs during neurulation and craniofacial patterning. Interestingly, NELF complex (containing WHSC2/NELF-A), has been shown to stabilize transcription of fatty acid oxidation-related genes (Pan et al., 2014)[66], which would suggest dual-depletion of these in areas where they are typically enriched (Fig.Figure 2) may greatly impact metabolic homeostasis. This could be especially damaging in the context of the multipotent CNCs, as metabolism is increasingly demonstrated to perform a commanding role in determination of cell fate (Shyh-Chang et al., 2013; Sperber et al., 2015; Mathieu and Ruohola-Baker, 2017; Perestrelo et al., 2018).

TACC3 is predominantly known as a microtubule regulator. Originally characterized as an essential centrosome adapter during cell division (Gergely et al., 2000; Piekorz et al., 2002), its manipulation was more recently shown to impact microtubule plus-end growth in interphase cells and specifically CNCs (Nwagbara et al., 2014). It has also demonstrated effects on cytoskeletal mechanics during one form of embryonic cell motility, axon outgrowth and guidance signal response (Nwagbara et al., 2014; Erdogan et al., 2017). Its significant dysregulation in metastatic cancers (Ha et al., 2013a,b; Li et al., 2017), and roles in mitotic spindle organization (Albee and Wiese, 2008; Cheeseman et al., 2011; Nixon et al., 2015; Burgess et al., 2018) may allude to additional functions in cytoskeletal coordination of either CNC proliferation or motility, but this remains unexplored. Altogether, it is clear that our current knowledge of how these genes ultimately contribute to embryonic development is lacking, and a basic cell biological examination of WHS-associated gene function within a developmental context is necessary for a better mechanistic understanding of WHS etiology.

Finally, it will also be essential to explore how these genes ultimately synergistically or epistatically regulate WHS pathology. To this aim, our model provides the unique advantage of titratable, rapid, and inexpensive combinatorial depletion of numerous genes, and an intuitive next step will be to perform depletions in tandem that would mirror the genetic perturbations identified from both typical and atypical case studies of WHS. Altogether, our current and ongoing work suggests significant roles for numerous 4p16.3 genes as potent effectors of neural crest-derived tissues and craniofacial morphogenesis.

## Materials and Methods

### *Xenopus* Husbandry

Eggs obtained from female *Xenopus laevis* were fertilized *in vitro*, dejellied and cultured at 13–22°C in 0.1X Marc’s modified Ringer’s (MMR) using standard methods (Sive et al., 2010). Embryos received injections of exogenous mRNAs or antisense oligonucleotide strategies at the two- or four- cell stage, using four total injections (1 injection per blastomere in 4-cell, 2 injections per blastomere in 2-cell) performed in 0.1X MMR media containing 5% Ficoll. Embryos were staged according to Nieuwkoop and Faber (1994). All experiments were approved by the Boston College Institutional Animal Care and Use Committee and were performed according to national regulatory standards.

### Immunostaining

Whole-mount immunostaining was carried out using mouse anti-acetylated tubulin (Sigma, St. Louis, MO, United States T7451, 1:500), with goat anti-mouse Alexa Fluor 488 (Invitrogen, 1:1000) as a secondary antibody. 5 dpf embryos were fixed in 4% paraformaldehyde in PBS for 1 h, rinsed in PBS and gutted to reduce autofluorescence. Embryos were processed for immunoreactivity by incubating in 3% bovine serum albumin, 1% Triton-X 100 in PBS for 2 h, then incubated in antibodies (4°C, overnight). Embryos were cleared in 1% Tween-20 in PBS and imaged in PBS after removal of the skin dorsal to the brain. Images were taken using a Zeiss AxioCam MRc attached to a Zeiss SteREO Discovery.V8 light microscope. Images were processed in Photoshop (Adobe, San Jose, CA, United States). Area of the forebrain and midbrain were determined from raw images using the polygon area function in ImageJ (Schneider et al., 2012). Statistical significance was determined using a student’s paired *t*-test.

### Whole Mount *in situ* Hybridization

Embryos were fixed overnight at 4°C in a solution of 4% paraformaldehyde in phosphate-buffered saline (PBS), gradually dehydrated in ascending concentrations of methanol in PBS, and stored in methanol at -20°C for a minimum of 2 h, before *in situ* hybridization, which was performed on fixed embryos as previously described (Saint-Jeannet, 2017). After brief proteinase K treatment, embryos were bleached under white light in 1.8× saline-sodium citrate, 1.5% H_2_O_2_, and 5% (vol/vol) formamide for 20 min to 1 h before prehybridization. During hybridization, probe concentration was 0.5 μg/mL. The *tacc3* construct used for a hybridization probe was subcloned into the pGEM T-easy vector (Promega, Madison, WI, United States). The *Xenopus* *Twist* hybridization probe was a kind gift from Dr. Dominique Alfandari (University of Massachusetts at Amherst, MA, United States), which was subcloned into the pCR 2.1TOPO vector (AddGene, Cambridge, MA, United States). The template for making an antisense probe for *letm1* was PCR amplified from the reverse transcribed cDNA library, using primer set (5′- CATGGCTTCCGACTCTTGTG, CTAGCTAATACGACTCACTATAGGGCTACAGATGGTACAG AGG-3′), then subcloned into the pCS2 vector (AddGene, Cambridge, MA, United States). Templates for *whsc1* and *whsc2* antisense probes were PCR amplified from ORFeomes (European Xenopus Resource Center, United Kingdom) with the following primer sets: *Whsc1* forward 5′-CTCATATCCTCGGAAGTCCAGC-3′, *whsc1* backward 5′-CTAGCTAATACGACTCACTATAGGACCATACAACATCTCC AACAG-3′, *whsc2* forward 5′-CCTCCGTCATAGACAAC GTG-3′, and *whsc2* backward 5′-CTAGCTAATACGACTCA CTATAGGAGAGGAGTTGTTGTGTCCAG-3′; these products were cloned into the pDONR223 vector (AddGene, Cambridge, MA, United States). The antisense digoxigenin-labeled hybridization probes were transcribed in vitro using the T7 MAXIscript kit. Embryos were imaged using a Zeiss AxioCam MRc attached to a Zeiss SteREO Discovery.V8 light microscope. Images were processed in Photoshop (Adobe, San Jose, CA, United States).

### Depletion

Morpholino antisense oligonucleotides (MO) were used to target WHS related genes. *Whsc2* and *tacc3* MOs targeted the translation start site of *Xenopus laevis* *whsc2* (5-TGTCACTATCCCTCATAGACGCCAT-3) and *tacc3* (5-AGTTGTAGGCTCATTCTAAACAGGA3), respectively. *Whsc1* MO targeted the intron exon boundary of intron 5 of *Xenopus laevis* *whsc1* (5-TGCGTTTTCATGTTTACCAGAGTCT-3) and *letm1* MO targeted the intron exon boundary of intron 1 of *Xenopus laevis* *letm1*(5-ATGACACACAAGTGCTACTTACCCT-3). These WHS gene specific MOs, or standard control MO (5-CCTCTTACCTCAGTTACAATTTATA-3) (purchased from Gene Tools, LLC, Philomath, OR, United States), were injected into two-to-four cell stage embryos (10–30 ng/embryo).

Knockdown of Whsc2 and Tacc3 were assessed by Western blot (Supplementary Figure S4). Embryos at stage 35 were lysed in buffer (50 mM Tris pH 7.5, 5% glycerol, 0.2% IGEPAL, 1.5 mM MgCl2, 125 mM NaCl, 25 mM NaF, 1 mM Na3VO4, 1 mM DTT, supplemented with Complete Protease Inhibitor Cocktail with EDTA, Roche). Blotting for Whsc2 was carried out using mouse monoclonal antibody to Whsc2 (Abcam, ab75359, dilution 1:3,000). *Tacc3* start site MO was validated as previously described (Monsoro-Burq, 2015). Detection was by chemiluminescence using Amersham ECL Western blot reagent (GE Healthcare BioSciences, Pittsburg PA, United States). The bands were quantified by densitometry using ImageJ (Schneider et al., 2012).

*Whsc1* and *letm1* splice site MOs were validated through a reverse transcriptase polymerase chain reaction (rt PCR). Total RNA was extracted by homogenizing embryos 48hrs post fertilization in Trizol. RNA purification was performed according to the Qiagen RNA purification protocol. A phenol:chloroform extraction was performed followed by ethanol precipitation. cDNA was synthesized using SuperScript II Reverse Transcriptase. PCR was performed in a Mastercycler using HotStarTaq following the Qiagen PCR protocol. Primers for *letm1* were as follows; forward 5′-GTACGAGGCTGTGTGCTGAG-3′ and backward 5′-CGGTTTCCACTTCGCTGACG -3′. Primers for *whsc1* were as follows; forward 5′-GTCGTACAAGAGAAGACGAGTG-3(’ and backward 5(’- GTCAGTGAAGCAGGAGAAGAAC- 3(’. Band intensity was measured using densitometry in ImageJ (Burgess et al., 2018; Supplementary Figure S4).

Rescue experiments were performed with exogenous mRNAs co-injected with their corresponding MO strategies. *Xenopus* ORFs for *whsc1* and *whsc2* were purchased from EXRC and gateway-cloned into pCSF107mT-GATEWAY-3′-LAP tag (Addgene plasmid #67618, a generous gift from Todd Stunkenberg). A complete coding sequence of *X. tropicalis* *letm1* was purchased from Dharmacon (Lafayette, CO, United States) then subcloned into pCS2+ EGFP vector. Plasmid for TACC3 cloned into pET30a was a kind gift from the Richter lab (University of Massachusetts Medical School, Worcester, MA, United States), which was subcloned into pCS2. As a start-site MO was utilized to block *tacc3* translation, a MO-resistant exogenous mRNA was generated by creating conserved mutations in the first 7 codons. Rescue concentrations are described in Supplementary Figure S3.

### Cartilage Staining

At 6 dpf, *Xenopus* embryos were anesthetized with benzocaine and fixed in cold 4% paraformaldehyde in PBS and were left at 4°C overnight. Alcian Blue staining of embryos was performed based on the Harland Lab protocol. Before ethanol dehydration, embryos were bleached under white light in 1.8x saline-sodium citrate, 1.5% H2O2, and 5% (vol/vol) formamide for 30 min. Embryos were imaged in PBS, using a Zeiss AxioCam MRc attached to a Zeiss SteREO Discovery.V8 light microscope. Images were processed in Photoshop (Adobe, San Jose, CA). Analysis of cartilage structures was performed in ImageJ utilizing the polygon, area, and line functions (Schindelin et al., 2012). Measurements included (1) Total cartilage area measured as the area of the cartilage from the base of the branchial arches, along either side of cartilage structure, and around the infracostal cartilage. (2) Average ceratohyal cartilage area (see outlined cartilage in Figure 4). (3) Branchial arch width was determined by measuring the width of the branchial arch region at the widest point. (4) Ceratohyal cartilage width was determined using the line function at the widest point on the ceratohyal cartilage. Differences were analyzed by student unpaired *t*-test.

### Quantifying Craniofacial Shape and Size

Stage 40 embryos (66 hpf) were fixed in 4% paraformaldehyde in PBS overnight at 4°C. A razor blade was used to make a cut bisecting the gut to isolate the head. Isolated heads were mounted in small holes in a clay-lined dish containing PBS. The faces were imaged using a Zeiss AxioCam MRc attached to a Zeiss SteREO Discovery. V8 light microscope. ImageJ (Schindelin et al., 2012) software was used to perform craniofacial measurements. These measurements included the: (1) intercanthal distance, or the distance between the eyes, (2) face height, or the distance between the top of the eyes and the top of the cement gland at the midline, (3) midface angle, the angle created by drawing lines from the center of one eye, to the dorsal midline of the mouth, to the center of the other eye, and (4) midface area, the area measured from the top of the eyes to the cement gland encircling the edges of both eyes (see Supplementary Figure S3). For all facial measurements, Student’s unpaired *t*-tests were performed between KD embryos and control MO injected embryos to determine statistical relationships. Protocol was lightly adapted from Kennedy and Dickinson (2014).

### Half Embryo Injections

Half knockdowns were performed at the two-cell stage; *X. laevis* embryos were unilaterally injected two times with both WHS gene-specific MO and a GFP mRNA construct. Half the quantity of morpholino was injected per embryo as compared to full bilateral knockdowns. The other blastomere was injected with a control MO at the same dose. Embryos were raised in 0.1X MMR through neurulation, at which point they were sorted based on left/right fluorescence. In order to complete pharyngeal arch visualization, embryos were fixed between stage 25–30 and whole-mount *in situ* hybridization for *twist* was performed according to the previously described procedure. For brain morphology analysis, embryos were fixed 6 dpf and prepared for alpha-tubulin immunostaining.

Analysis of pharyngeal arches from *in situ* experiments was performed on lateral images in ImageJ (Schneider et al., 2012). Measurements were taken to acquire: (1) Arch area: the area of individual *twist* labeled streams within the PA, determined using the polygon tool. (2) Arch length: The length of the distance between the top and bottom of each *twist* labeled CNC stream. (3) Arch migration was determined using the line function, as measured from the ventral most part of the *twist* signal to the neural tube. Statistical significance was determined using a student’s paired *t*-test in Graphpad (Prism).

### Neural Crest Explants, Imaging, and Analysis

A very helpful and thorough guide to neural crest isolation has been described previously (Alfandari et al., 2001; Milet and Monsoro-Burq, 2014).

We offer only minor modifications here. Stage 18 embryos were placed in modified DFA solution (53 mM NaCl, 11.7 mM Na2CO3, 4.25 mM K Gluc, 2 mM MgSO4, 1 mM CaCl2, 17.5 mM Bicine, with 50 μg/mL Gentamycin Sulfate, pH 8.3), before being stripped of vitelline membranes and imbedded in clay with the anterior dorsal regions exposed. Skin was removed above the neural crest using an eyelash knife, and neural crest explants were dissected out. Explants were rinsed, and plated on fibronectin-coated coverslips in imaging chambers filled with fresh DFA. Tissues were allowed to adhere 45 min before being moved to the microscope for time lapse imaging of CNC motility.

Microscopy was performed on a Zeiss Axio Observer inverted motorized microscope with a Zeiss 20x N-Achroplan 0.45 NA phase contrast lens, using a Zeiss AxioCam camera controlled with Zen software. Images were collected using large tiled acquisitions to capture the entire migratory field. Eight to ten explants, from both control and experimental conditions were imaged at a 6 min interval, for 3 h. Data was imported to Fiji (Schindelin et al., 2012), background subtracted, and cropped to a uniform field size. Migration tracks of individual cells were collected using automated tracking with the Trackmate plug-in (Tinevez et al., 2017). Mean speeds were imported to Prism (Graphpad), and compared between conditions using unpaired *t*-tests. Three independent experiments were performed for each experimental condition.

## Author’s Note

Wolf-Hirschhorn Syndrome (WHS), a developmental disorder caused by small deletions on chromosome four, manifests with pronounced and characteristic facial malformation. While genetic profiling and case studies provide insights into how broader regions of the genome affect the syndrome’s severity, we lack a key component of understanding its pathology; a basic knowledge of how individual WHS-affected genes function during development. Importantly, many tissues affected by WHS derive from shared embryonic origin, the cranial neural crest. This led us to hypothesize that genes deleted in WHS may hold especially critical roles in this tissue. To this end, we investigated the roles of four WHS-associated genes during neural crest cell migration and facial patterning. We show that during normal development, expression of these genes is enriched in migratory neural crest and craniofacial structures. Subsequently, we examine their functional roles during facial patterning, cartilage formation, and forebrain development, and find that their depletion recapitulates features of WHS craniofacial malformation. Additionally, two of these genes directly affect neural crest cell migration rate. We report that depletion of WHS-associated genes is a potent effector of neural crest-derived tissues, and suggest that this explains why WHS clinical presentation shares so many characteristics with classic neurochristopathies.

## Ethics Statement

All experiments were approved by the Boston College Institutional Animal Care and Use Committee and were performed according to national regulatory standards.

## Author Contributions

LL, AM, and EB contributed conception and design of the study. EB, AM, RC, SK, MS, and SL performed the experiments and analyzed the data. EB wrote the first draft of the manuscript. All authors contributed to manuscript revision, read and approved the submitted version.

## Conflict of Interest Statement

The authors declare that the research was conducted in the absence of any commercial or financial relationships that could be construed as a potential conflict of interest.

## Supplementary Material

FIGURE S1 Expression patterns for WHS related genes across early development. *In situ* hybridization utilized **(A–F)** antisense mRNA probe to *whsc1*, **(G–L)** full-length antisense mRNA probe to *whsc2*, **(M–R)** full-length antisense mRNA probe to *letm1*, and (S-X) 1 kb partial-length antisense mRNA probe to *tacc3*. Embryos shown at blastula stage **(A,G,M,S)**, in dorsal view at stage 16–20 **(B,H,N,T)**, in lateral view at stage 20–25 **(C,I,O,U)**, detail of lateral anterior region at stage 35 **(D,J,P,V)**, and in both lateral and dorsal views from stages 39–42 (**E,K,Q,W** and **F,L,R,X**). **(Y)** *In situ* hybridization probes generated against sense strands of WHS gene mRNAs, shown at stage 25. Brown coloration **(S,T)** is unbleached pigment, unrelated to *in situ* hybridization staining. Scalebar is 250 μm.bryos were anesthetized with benzocaine.

FIGURE S2 Validation of WHS related MOs. **(A,B)** Gel of polymerase chain reaction (PCR) that shows injection of 10 ng MO targeted to *whsc1* mRNA causes a greater than 80% reduction at 2 dpf. **(C,D)** Injection of 20 ng of a MO targeted against *letm1* causes an 55% decrease in *letm1* mRNA 2 dpf. **(E,F)** Western blot showing 10 ng injection of a MO targeted against *whsc2* results in a greater than 50% reduction in Whsc2 protein by 2 dpf. **(G,H)** Western blot showing 40 ng of a MO targeted against *tacc3* results in 22% reduction. Bar graphs **(B,D,F,H)** depict densitometry of gels **(A,C)** or blot **(E,G)** shown, but is consistent across triplicate results.

FIGURE S3 Demonstration of measurement schemes for craniofacial morphology. Measurements for Figure 3 and Supplementary Figure S4 were performed as indicated. **(A)** Facial width was measured from the center of each eye. **(B)** Facial height was determined at the midline, from the top of the cement gland to the top of the eyes. **(C)** Midface angle was measured from the top of the cement gland to the center of each eye. **(D)** Facial area was measured as the space encapsulated within the perimeter of each eye. Adapted from Kennedy and Dickinson (2014).

FIGURE S4 Craniofacial defects caused by WHS-associated gene KD are rescued by co-injection of exogenous mRNA co-expression. Facial widths from control, depletion, or rescue strategies were measured in tadpoles (st. 40). Row 1: Embryos injected with **(A)** control MO (*n* = 17), **(B)** 10 ng *whsc1* MO (*n* = 14), or **(C)** 10 ng of *whsc1* MO and 250 pg of *whsc1* exogenous mRNA. **(D)** Comparisons of facial width showed an 8.76% increase in facial width with Whsc1 KD, which was rescued by *whsc1* mRNA co-injection. Row 2: Embryos injected with **(E)** control MO (*n* = 21), **(F)** 10 ng *whsc2* MO (*n* = 17), or **(G)** both 10ng of *whsc2* MO and 250 pg of *whsc2* mRNA (*n* = 19). **(H)** Whsc2 knockdown caused an 8.37% reduction in facial width, which was rescued by exogenous *whsc2* mRNA co-injection. Row 3: Embryos injected with **(I)** control MO (*n* = 10), **(J)** 20 ng of *letm1* MO, or **(K)** 20 ng *letm1* MO and 1500 pg of *letm1* mRNA (*n* = 11). **(L)** KD of Letm1 caused a 14.95% decrease in facial width, and was rescued by co-injection of exogenous *letm1* mRNA. Row 4: Embryos injected with **(M)** control MO (*n* = 9), **(N)** 20 ng of *tacc3* MO (*n* = 18), or O) 20 ng of *tacc3* morpholino and 1000 pg of *tacc3* mRNA (*n* = 16). **(P)** Tacc3 KD resulted in a 9.01% decrease in facial width, and was rescued by *tacc3* mRNA co-injection. Significance determined using a student’s unpaired *t*-test. ^∗∗^*P* < 0.01, ^∗^*P* < 0.05, n.s., not significant. Scalebar is 250μm.

FIGURE S5 Half embryo knockdown can be utilized for analysis of brain morphology and neural crest cell migration *in vivo.* **(A)** At the 2-cell stage, a single blastomere is injected with WHS-associated gene MOs and exogenous eGFP mRNA. After neurulation (approx. stage 21), embryos are sorted based on left or right eGFP fluorescence, to determine side of depletion. To examine neural crest cell size, migration and morphology embryos were fixed between stage 25–30, and *in situ* hybridization was performed using *twist* anti-sense probe. To characterize brain morphology, embryos were raised to st. 47, and fixed and labeled with alpha-tubulin antibody, a neuronal marker, and Alexa-488 secondary. **(B–D)** Control MO does not significantly impact brain size, compared to non-injected hemispheres (a paired internal control).
