# Corrigendum: Wolf-Hirschhorn Syndrome-Associated Genes Are Enriched in Motile Neural Crest Cells and Affect Craniofacial Development in *Xenopus laevis*

**DOI:** 10.3389/fphys.2020.644596

**Published:** 2021-02-16

**Authors:** Alexandra Mills, Elizabeth Bearce, Rachael Cella, Seung Woo Kim, Megan Selig, Sangmook Lee, Laura Anne Lowery

**Affiliations:** Biology Department, Boston College, Chestnut Hill, MA, United States

**Keywords:** craniofacial development, developmental disorders, Wolf-Hirschhorn Syndrome, WHSC1, WHSC2, LETM1, TACC3, neural crest

In the original article, Figure 6 panel D (left) was mislabeled. It has been corrected to read Whsc1 KD. In Figure 7, panel E was mislabeled. It has been corrected to read Whsc2 KD.

**Figure 6:**
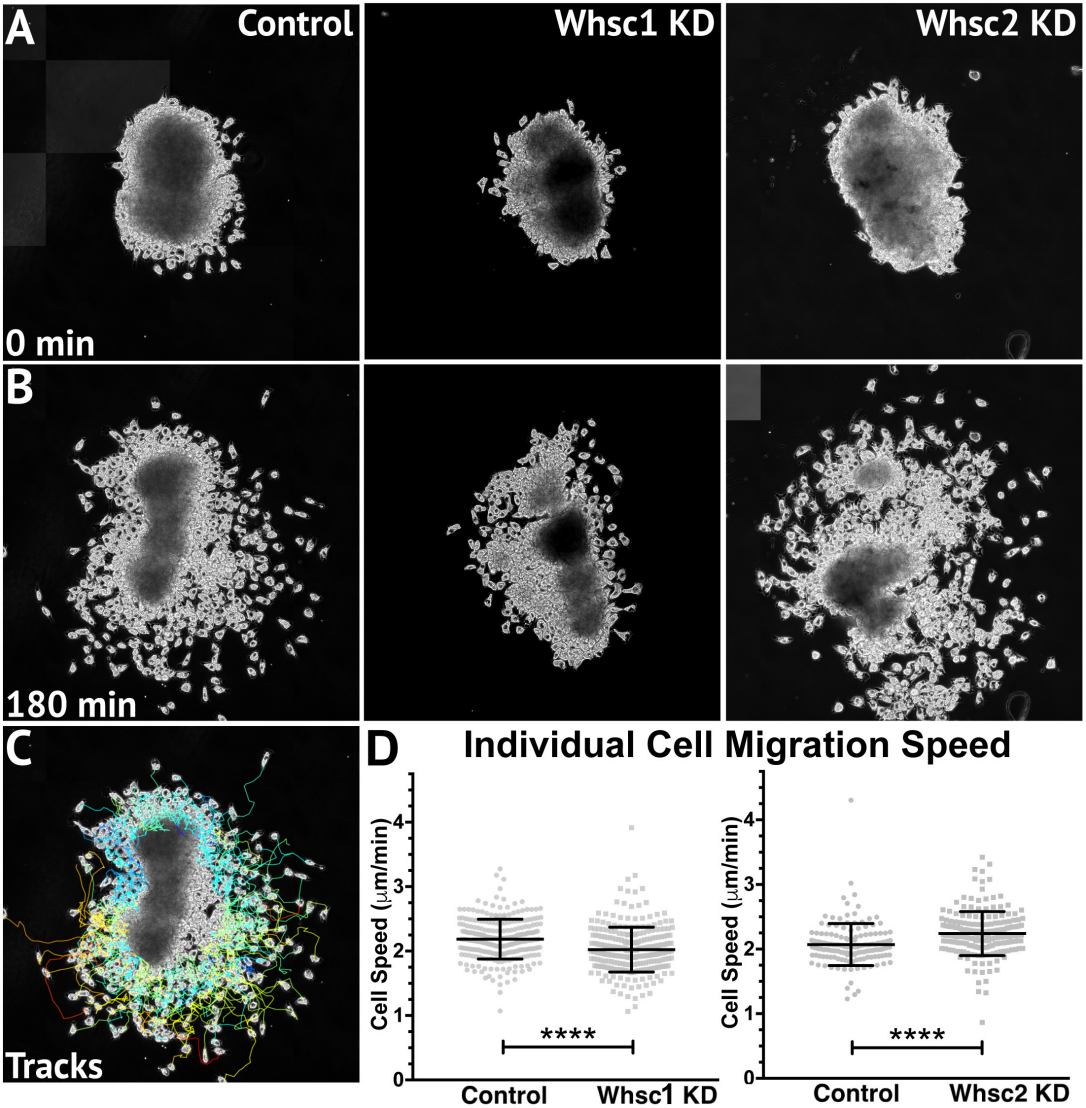
Whsc1 manipulation alters CNC migration speeds *in vitro*. Dissected CNC explants from control, Whsc1 KD, or Whsc2 KD embryos were plated on fibronectin-coated coverslips, allowed to adhere and begin migration, and imaged for 3 h using 20× phase microscopy. **(A)** Representative explants at initial timepoint (0 min). **(B)** Explants after 3 h migration time. **(C)** Representative tracks generated by FiJi Trackmate plug-in. **(D)** Mean track speeds of Whsc1 or Whsc2 KD explants compared to their controls. (Explants quantified: 3–4 explants from control and KD embryos were plated for each experiment, explants with neural or epithelial contaminant were excluded from analysis. Three separate experiments were performed for each depletion. Whsc1 controls: 272 cells, 9 explants. Whsc1 KD: 282 cells, 9 explants. Whsc2 controls: 151 cells, 12 explants. Whsc2 KD: 195 cells, 8 explants.) ****P < 0.0001, n.s., not significant. Scalebar is 250 μm.

**Figure 7:**
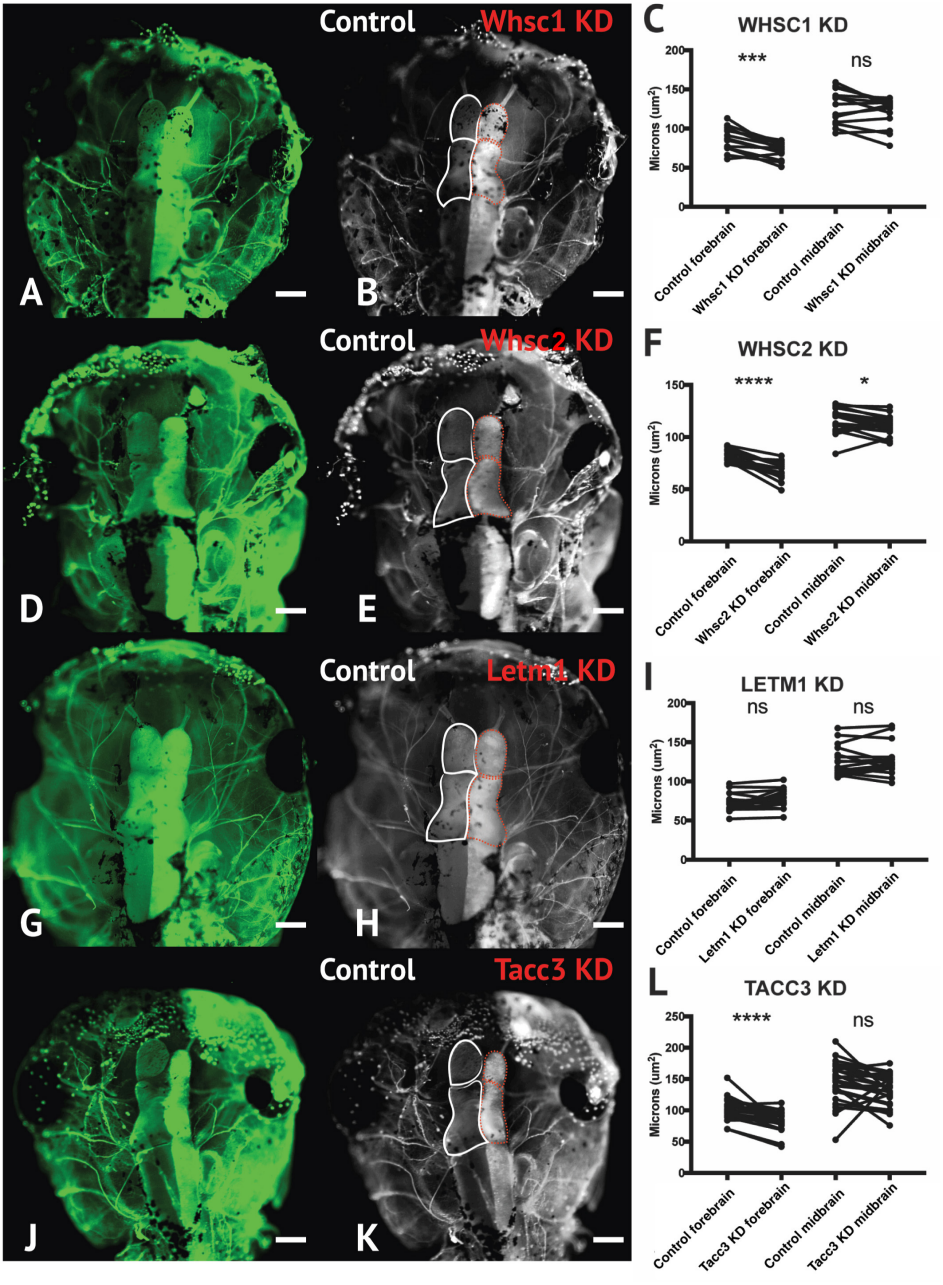
Whsc1, whsc2, and tacc3 facilitate normal forebrain development. **(A,B,D,E,G,H,J,K)** Dorsal view of *X. laevis* half-embryo gene depletions (6 days post-fertilization), following alpha-tubulin immunolabeling to highlight nervous system. **(B,E,H,K)** Dorsal view of embryos with superimposed outlines of forebrain and midbrain structures. Internal control is on left (white), depleted side is on right (dashed red). (Alpha-tubulin staining is bilateral; exogenous eGFP on KD side persisted in embryos shown, causing a unilaterally enriched green signal.) **(C,F,I,L)** Area of forebrain and midbrain. Whsc1 KD reduced forebrain area by 17.65%. Whsc2 KD reduced forebrain area by 17.33% and midbrain area by 4.14%. Letm1 KD caused no significant change in brain size. Tacc3 KD caused a 16.05% decrease in forebrain area. Significance determined using a student's paired *t*-test. (Embryos quantified: Whsc1 KD = 14, Whsc2 KD = 18, Letm1 KD = 12, Tacc3 KD = 26.) ****P < 0.0001, ***P < 0.001, *P < 0.05, n.s., not significant. Scalebar is 250 μm.

