# Supplementary figures and images for: Wolf-Hirschhorn Syndrome-Associated Genes Are Enriched in Motile Neural Crest Cells and Affect Craniofacial Development in Xenopus laevis
Source: Front Physiol. 2019 Apr 12;10:431. doi: 10.3389/fphys.2019.00431 (PMC6474402; doi:10.3389/fphys.2019.00431)

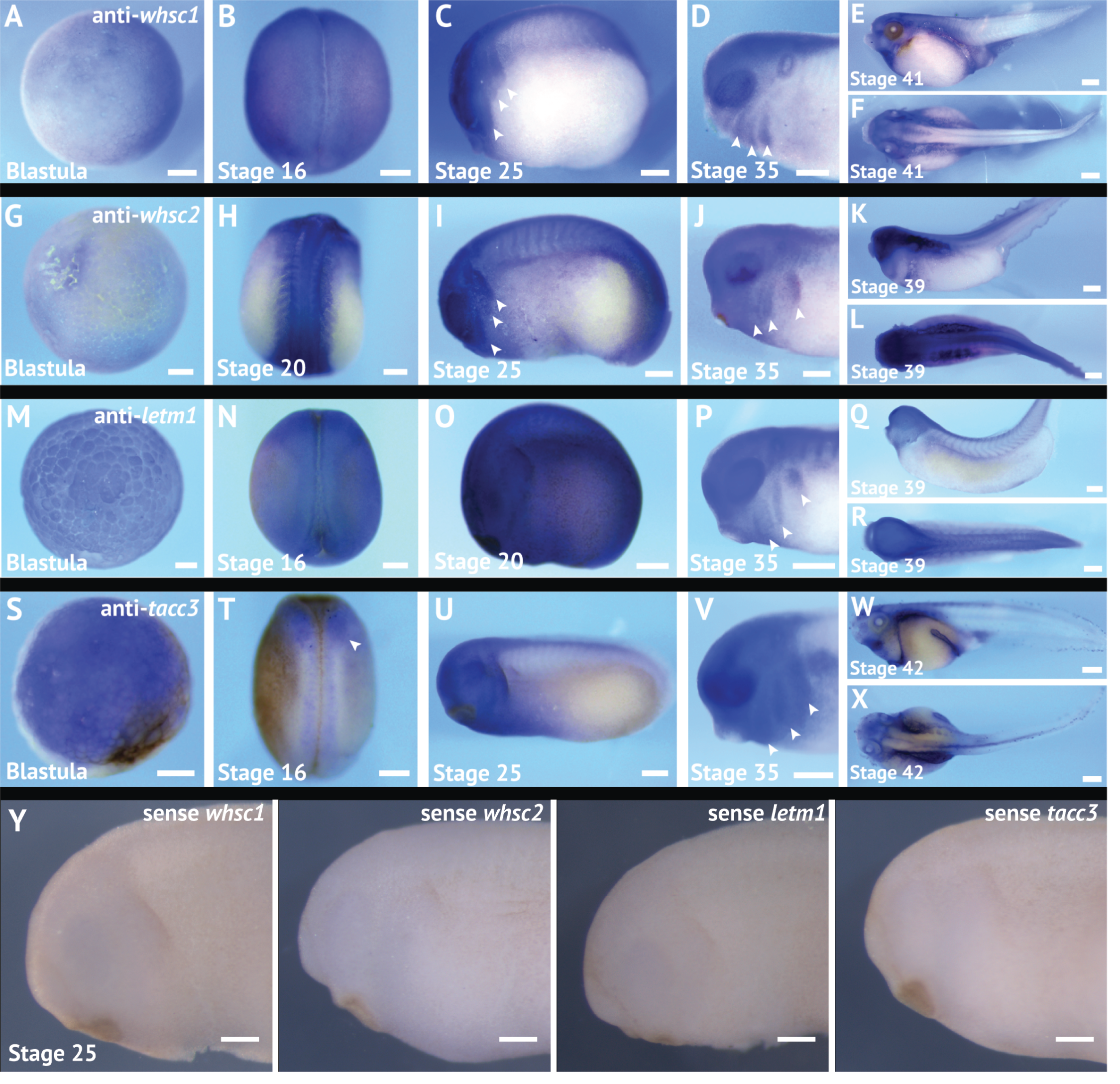

Supplement: FIGURE S1 — Expression patterns for WHS related genes across early development. In situ hybridization utilized (A–F) antisense mRNA probe to whsc1, (G–L) full-length antisense mRNA probe to whsc2, (M–R) full-length antisense mRNA probe to letm1, and (S-X) 1 kb partial-length antisense mRNA probe to tacc3. Embryos shown at blastula stage (A,G,M,S), in dorsal view at stage 16–20 (B,H,N,T), in lateral view at stage 20–25 (C,I,O,U), detail of lateral anterior region at stage 35 (D,J,P,V), and in both lateral and dorsal views from stages 39–42 (E,K,Q,W and F,L,R,X). (Y) In situ hybridization probes generated against sense strands of WHS gene mRNAs, shown at stage 25. Brown coloration (S,T) is unbleached pigment, unrelated to in situ hybridization staining. Scalebar is 250 μm.bryos were anesthetized with benzocaine. [file Image_1.TIF]

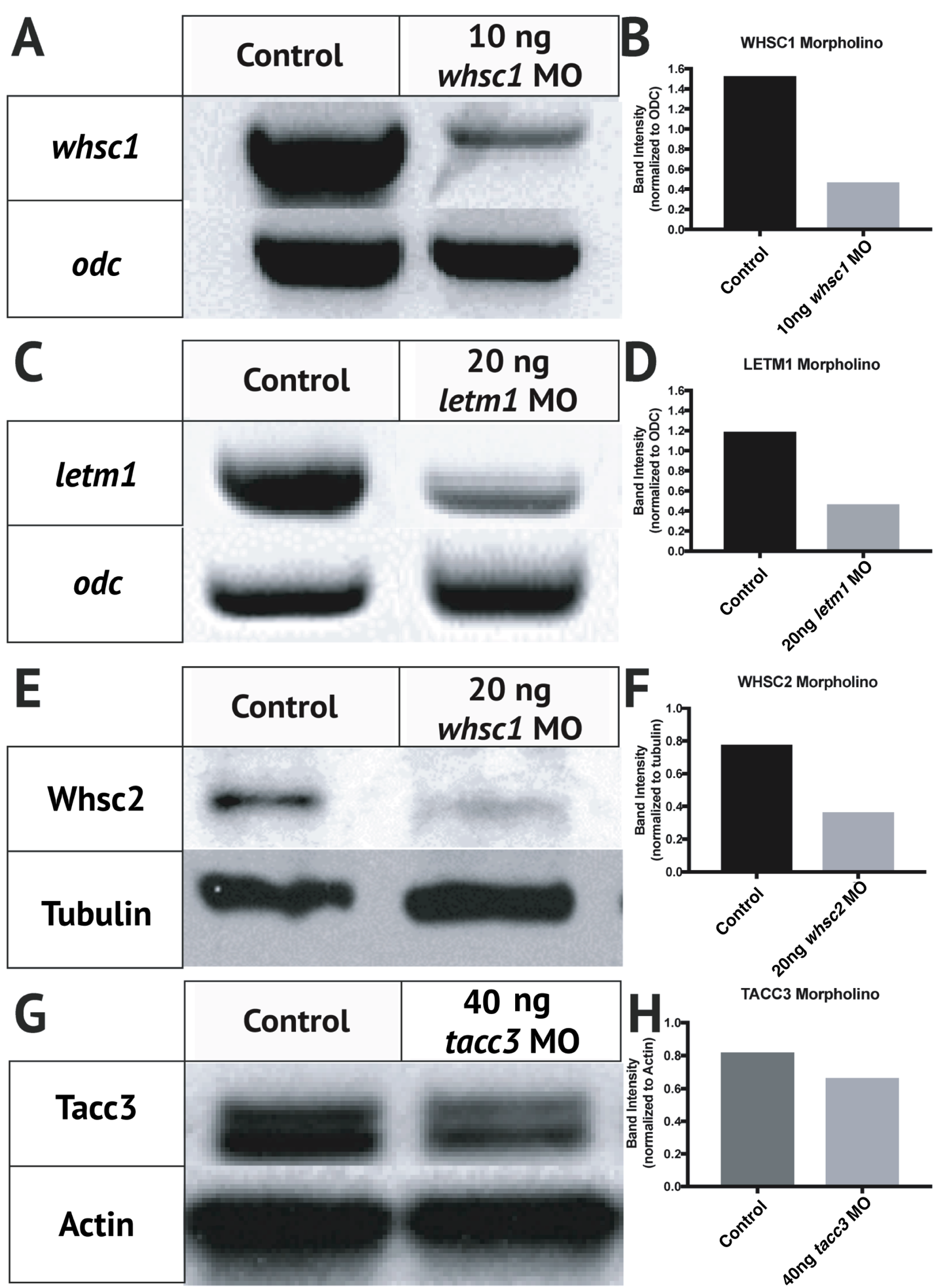

Supplement: FIGURE S2 — Validation of WHS related MOs. (A,B) Gel of polymerase chain reaction (PCR) that shows injection of 10 ng MO targeted to whsc1 mRNA causes a greater than 80% reduction at 2 dpf. (C,D) Injection of 20 ng of a MO targeted against letm1 causes an 55% decrease in letm1 mRNA 2 dpf. (E,F) Western blot showing 10 ng injection of a MO targeted against whsc2 results in a greater than 50% reduction in Whsc2 protein by 2 dpf. (G,H) Western blot showing 40 ng of a MO targeted against tacc3 results in 22% reduction. Bar graphs (B,D,F,H) depict densitometry of gels (A,C) or blot (E,G) shown, but is consistent across triplicate results. [file Image_2.TIF]

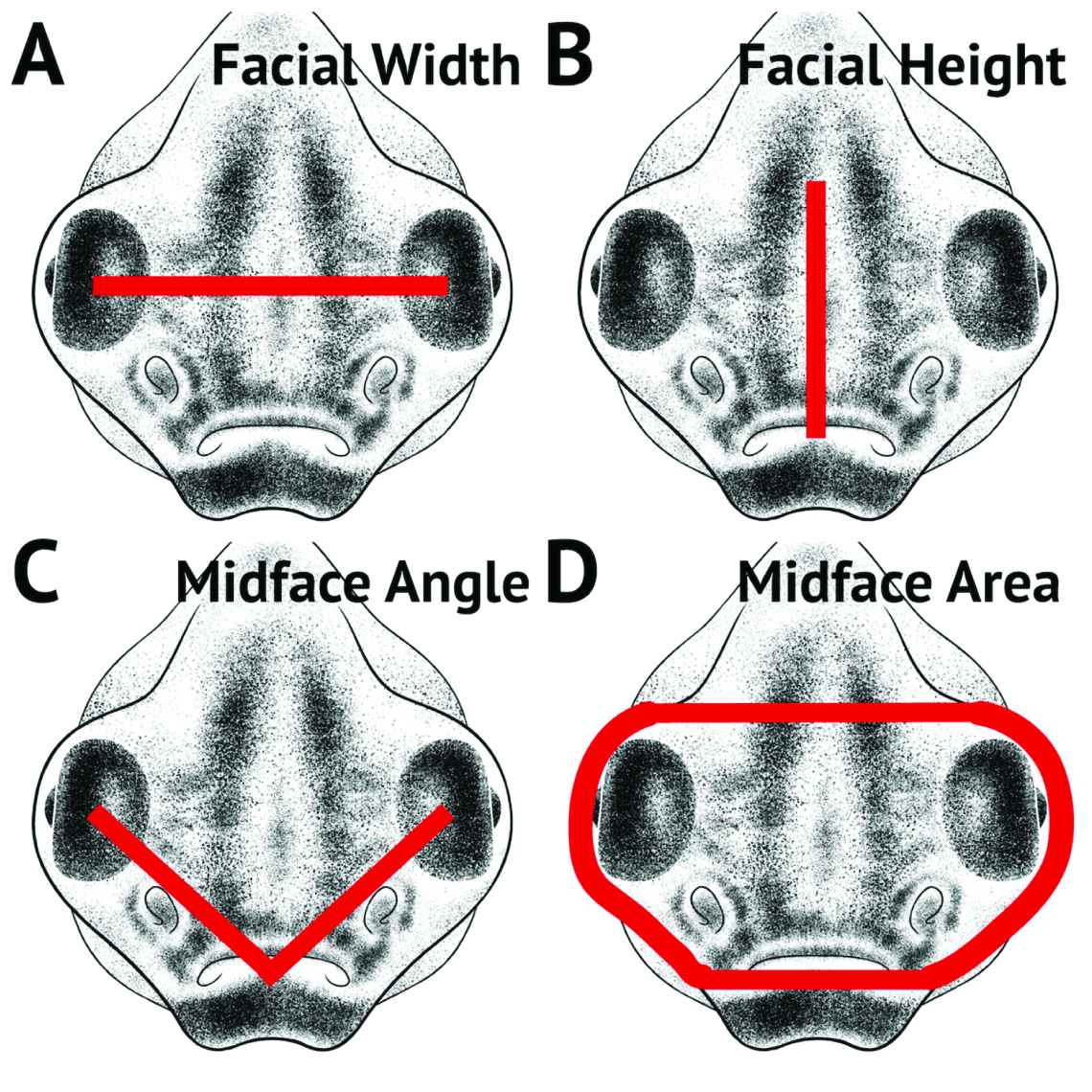

Supplement: FIGURE S3 — Demonstration of measurement schemes for craniofacial morphology. Measurements for Figure 3 and Supplementary Figure S4 were performed as indicated. (A) Facial width was measured from the center of each eye. (B) Facial height was determined at the midline, from the top of the cement gland to the top of the eyes. (C) Midface angle was measured from the top of the cement gland to the center of each eye. (D) Facial area was measured as the space encapsulated within the perimeter of each eye. Adapted from Kennedy and Dickinson (2014). [file Image_3.TIF]

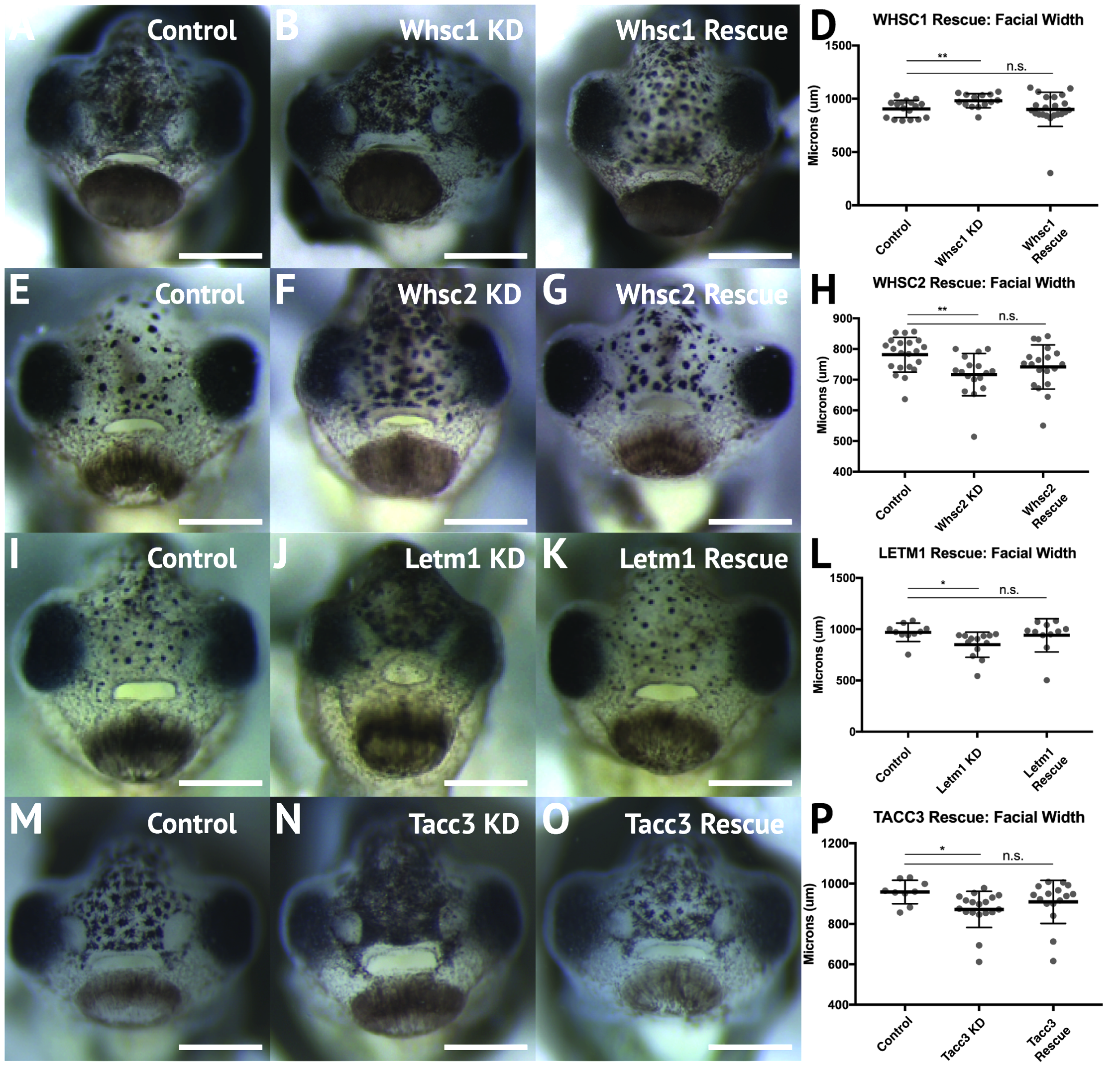

Supplement: FIGURE S4 — Craniofacial defects caused by WHS-associated gene KD are rescued by co-injection of exogenous mRNA co-expression. Facial widths from control, depletion, or rescue strategies were measured in tadpoles (st. 40). Row 1: Embryos injected with (A) control MO (n = 17), (B) 10 ng whsc1 MO (n = 14), or (C) 10 ng of whsc1 MO and 250 pg of whsc1 exogenous mRNA. (D) Comparisons of facial width showed an 8.76% increase in facial width with Whsc1 KD, which was rescued by whsc1 mRNA co-injection. Row 2: Embryos injected with (E) control MO (n = 21), (F) 10 ng whsc2 MO (n = 17), or (G) both 10ng of whsc2 MO and 250 pg of whsc2 mRNA (n = 19). (H) Whsc2 knockdown caused an 8.37% reduction in facial width, which was rescued by exogenous whsc2 mRNA co-injection. Row 3: Embryos injected with (I) control MO (n = 10), (J) 20 ng of letm1 MO, or (K) 20 ng letm1 MO and 1500 pg of letm1 mRNA (n = 11). (L) KD of Letm1 caused a 14.95% decrease in facial width, and was rescued by co-injection of exogenous letm1 mRNA. Row 4: Embryos injected with (M) control MO (n = 9), (N) 20 ng of tacc3 MO (n = 18), or O) 20 ng of tacc3 morpholino and 1000 pg of tacc3 mRNA (n = 16). (P) Tacc3 KD resulted in a 9.01% decrease in facial width, and was rescued by tacc3 mRNA co-injection. Significance determined using a student’s unpaired t-test. ∗∗P < 0.01, ∗P < 0.05, n.s., not significant. Scalebar is 250μm. [file Image_4.TIF]

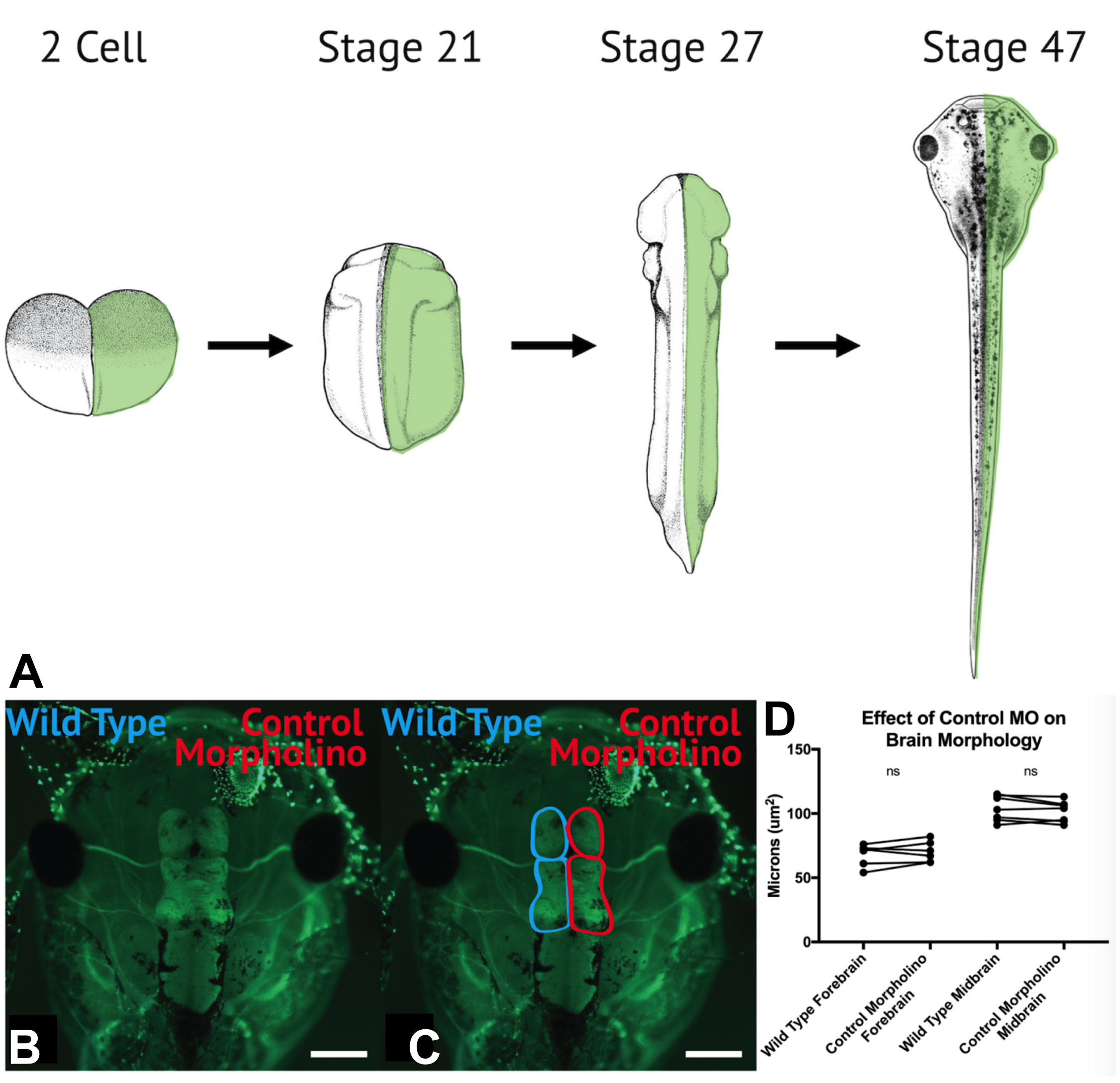

Supplement: FIGURE S5 — Half embryo knockdown can be utilized for analysis of brain morphology and neural crest cell migration in vivo. (A) At the 2-cell stage, a single blastomere is injected with WHS-associated gene MOs and exogenous eGFP mRNA. After neurulation (approx. stage 21), embryos are sorted based on left or right eGFP fluorescence, to determine side of depletion. To examine neural crest cell size, migration and morphology embryos were fixed between stage 25–30, and in situ hybridization was performed using twist anti-sense probe. To characterize brain morphology, embryos were raised to st. 47, and fixed and labeled with alpha-tubulin antibody, a neuronal marker, and Alexa-488 secondary. (B–D) Control MO does not significantly impact brain size, compared to non-injected hemispheres (a paired internal control). [file Image_5.TIF]
